# Direct estimation of the parameters of a delayed, intermittent activation feedback model of postural sway during quiet standing

**DOI:** 10.1371/journal.pone.0222664

**Published:** 2019-09-17

**Authors:** Kevin L. McKee, Michael C. Neale

**Affiliations:** Virginia Commonwealth University, Virginia Institute of Psychiatric and Behavioral Genetics, Richmond, Virginia, United States of America; Brandeis University, UNITED STATES

## Abstract

Human postural sway during quiet standing has been characterized as a proportional-integral-derivative controller with intermittent activation. In the model, patterns of sway result from both instantaneous, passive, mechanical resistance and delayed, intermittent resistance signaled by the central nervous system. A Kalman-Filter framework was designed to directly estimate from experimental data the parameters of the model’s stochastic delay differential equations with discrete dynamic switching conditions. Simulations showed that all parameters could be estimated over a variety of possible data-generating configurations with varying degrees of bias and variance depending on their empirical identification. Applications to experimental data reveal distributions of each parameter that correspond well to previous findings, suggesting that many useful, physiological measures may be extracted from sway data. Individuals varied in degree and type of deviation from theoretical expectations, ranging from harmonic oscillation to non-equilibrium Langevin dynamics.

## Introduction

Several previous studies have analyzed bodily sway patterns in quiet standing, and a variety of models have been proposed. In this study, we designed and tested a method of directly estimating the parameters of the Asai et al. [1] intermittent feedback control model of posture from experimental data. We begin with a brief review of prior models and the rationale for choosing a model of intermittent postural control (IPC). In the second section, we describe the current model in more detail and explain our framework for the estimation of its parameters. The third section describes simulation studies that tested the estimation capabilities of our framework when the data-generating parameters are known and the model is specified accurately. In the fourth section, we applied the model to experimental data and estimated sampling distributions for each parameter.

Observed trajectories of postural sway have largely been studied as a problem of stochastic behavior, though some studies have focused on its chaotic properties [2]. In this study, we too regarded postural sway as a random process subject to statistical analysis. The center of pressure (COP) on a force plate during quiet standing has been shown to exhibit the features of a bounded, random walk, or correlated noise [3]. Center of mass (COM) is one of the most common metrics of body sway but has to be inferred from other position and force metrics such as the COP [4]. For the small radius in which postural sway occurs, body tilt angle is nearly equivalent to COM and has likewise been used to develop models of posture [1].

Many authors have observed that sway follows a two-frequency oscillation scheme, with fast oscillations of the COP around a drifting center point [3, 5–7]. Collins and De Luca [3] regarded these patterns as a combination of short-term, open-loop system with long-term closed-loop control. Alternatively, the “rambling and trembling” hypothesis suggests that short-term tremors result from corrective, closed-loop feedback that is activated with deviation of the COP from the ground projection of the COM, which is itself allowed to drift [7].

Broadly, more recent debate over the control scheme of human balance has focused on two kinds of models: continuous and intermittent feedback controllers. Continuous control is exerted through a proportional-integral-derivative (PID) or closed-loop system often characterized by a second order linear differential equation, sometimes including delayed proportional and derivative feedback. For instance, Maurer and Peterka [8] tested a PID inverted pendulum model that distinguishes passive, instantaneous feedback from sources such as ankle joint stiffness, from delayed, active feedback from the central nervous system and subsequent muscular response. Others have argued that human postural movement is better described by intermittent feedback mechanisms due to a smaller reliance on process noise, reproduction of cyclical behavior over multiple timescales, more efficient energy expenditure, and greater robustness to disturbances and instability caused by delays in neural signaling [9]. Simulations [10] and reinforcement learning [11] have been used to show that an upright pendulum, taken as a simple model of the standing human body, can exhibit stability and the observed slow oscillation patterns as a result of learned, time-delayed, intermittent feedback.

Intermittent activation models have taken multiple forms. Gawthrop and Wang [12] initially proposed clock-driven muscular feedback, but later considered event-driven models [13]. Event-driven models are generally defined by a combination of stable and unstable manifolds in the phase space of the body’s position or angle. Gawthrop et al. [13] and Eurich and Milton [14] describe the behavior of systems with position-based thresholds that result in two stable equilibria. A model by Bottaro et al. [15] proposes boundary functions of both position and velocity that jointly determine probabilistic bursts of negative feedback. Asai et al. [1] reproduced a commonly observed double power-law structure in the PSD of sway [6] using similar control manifolds but with deterministic rules for sustained feedback activation. Their model requires only a simpler, Gaussian distribution of process noise with a smaller variance as compared to continuous PID models. Nomura et al. [16] showed that the same intermittent activation feedback model is capable of reproducing both chaotic and stochastic patterns that resemble human postural sway as a function of small hemodynamic perturbations, while continuous feedback models cannot.

A common method of estimating the parameters of each model is to simulate data that optimally resemble the experimental data. This is accomplished by varying parameters over iterations of simulation until resulting disparities on a set of key summary statistics have been minimized. Bottaro et al. [15] used the the Root Mean Square (RMS) of both the COP and COM series and each of its derivatives, unimodality of the series histogram, the length of largest oscillations calculated from zero crossings, and the PSD of the COP series. Maurer and Peterka [8] estimated parameters from observed data in a similar manner using mean velocity, RMS distance and velocity, spectral properties such as mean frequency, frequency dispersion, and total power. Asai et al. [1] used the double power law structure of the frequency spectrum as a criterion for the success of their model but did not demonstrate a direct empirical application. To obtain statistical information about estimated parameters, summary statistic methods have been combined with approximate Bayesian inference [17]. This method was used to acquire empirical posterior distributions of five out of the eight parameters of interest [18].

While the simulation approach is flexible for a wide range of model specifications and levels of complexity, it risks overlooking attributes of the data that do not have specific effects on the chosen summary statistics. Bottaro et al. [15] notes, for instance, that “The intermittent nature of the control process cannot be detected by global descriptors of the sway patterns, like the PSD of the COP, because they cannot distinguish between asymptotic and bounded stability”. Furthermore, the amplitude spectrum is invariant to reversal of the signal, giving identical results for potentially different mechanisms of variation. This is problematic when the system includes discontinuous dynamics, such as a sharp impulse followed by a more gradual decay. An alternative approach that better accounts for fine-grained sequential dependence is to estimate the structural parameters directly from the data using Kalman filtering or other iterative techniques. No simulation or descriptive statistics are necessarily used, rather the structural parameters are estimated by minimizing an objective function such as the squared prediction error, or by maximizing the likelihood of the data according to an expected noise distribution. The results obtained by this approach can be sensitive to the exact predictive mechanisms specified in the model, and post-hoc analyses of the estimates can be highly informative about the types and degrees of misspecification. Direct estimation (sometimes called *exact* estimation by comparison [19]) may be particularly useful when the dynamic structure cannot represented by any descriptive statistics with sufficient specificity. The Asai et al. [1] model of posture may present one such case in that it postulates dependence of the spectral power-law property upon nonlinear, physiological mechanisms of feedback control and their properties. Such properties include the delay in neural signaling, the sensitivity of feedback activation, and the strength of passive versus active corrective forces. Furthermore, the process noise distribution of the Asai et al. [1] model is a Gaussian process and thus accords with the statistical assumptions of the Kalman filter. A drawback of direct estimation is that a misspecified model is not guaranteed to result in any interpretable or accurate parameter estimates if the parameters are highly dependent. If the parameter estimates deviate significantly from their theorized values, we may nonetheless analyze the behaviors they imply and draw general inferences.

The aim of this study was to validate and apply a method of directly estimating parameters for event-driven control with specific focus on the popular intermittent control model by Asai et al. [1]. Validation of this analytic strategy will set a foundation for estimating the parameters of alternative models and more comprehensive comparisons. Following the validation study, we estimated empirical values of each parameter from publicly available COM data [20] and compared our results with theoretically expected values from the literature. We included two previously demonstrated covariates in our analysis, visual feedback and age, to attempt to replicate previous findings as further evidence for the validity of the model.

### Model

The intermittent postural control (IPC) model by Asai et al. [1] describes a tension between toppling torque due to gravity and a combination of active and passive resistance mechanisms. Passive resistance is proposed to come from leg stiffness and joint friction and is modeled with instantaneous relations between position, velocity, and acceleration. Active feedback control is proposed to arise from motor responses signaled by the central nervous system and is consequently delayed by about 190-210 ms [21].

The model is provided in terms of body tilt angle (*θ*) as follows:

$$\begin{array}{cc} I{\ddot{\theta }}_{t}= & mgh\theta_{t}-T, \end{array}$$ (1)

$$\begin{array}{ccc} T= & mghK\theta_{t}+B\dot{\theta_{t}}+mghf_{P}\left(\theta_{t-\tau }\right)+f_{D}\left({\dot{\theta }}_{t-\tau }\right)+\sigma w_{t}, & w_{t}∼N\left(0,1\right), \end{array}$$ (2)

where *I* is the rotational inertia, *m* is the body mass (kg), *g* is gravity (≈ 9.81*m*/*s*^2^), and *h* is the height of the COM. *T* includes all the terms representing mechanisms of resistance to the angular toppling force. *w*_*t*_ is a Gaussian, independent and identically distributed random variable accounting for stochastic variation in acceleration, with standard deviation *σ*. The total passive forces may be written as *mgh*(1 − *K*)*θ*_*t*_, as *K* is the percentage of the gravitational acceleration counteracted by passive resistance. While a certain definition of *B* is not given, its effects are non-trivial and an interpretation may be taken from the common use of the second-order damped oscillator equation, in which the velocity coefficient represents negative feedback due to friction. In this case, it may be regarded as a measure of ankle and knee joint friction.

The active control terms, *f*_*P*_ and *f*_*D*_, intermittently respond to *θ* on a time lag of *τ* ≈ 200 ms according to the conditions:

$$\begin{array}{cc} \text{if}\;\theta_{t-\tau }\left(s{\dot{\theta }}_{t-\tau }-\alpha \theta_{t-\tau }\right)>0,\text{and}\;\theta_{t-\tau }^{2}+{\left(s{\dot{\theta }}_{t-\tau }\right)}^{2}>r^{2} & \left\{ \begin{array}{c} f_{P}\left(\theta_{t-\tau }\right)=P\theta_{t-\tau }\\ f_{D}\left({\dot{\theta }}_{t-\tau }\right)=D{\dot{\theta }}_{t-\tau } \end{array}\text{(Active)},\right. \end{array}$$ (3)

$$\begin{array}{cc} \text{otherwise}\; & \left\{ \begin{array}{c} f_{P}\left(\theta_{t-\tau }\right)=0\\ f_{D}\left({\dot{\theta }}_{t-\tau }\right)=0 \end{array}\text{(Inactive)}\right. \end{array}$$ (4)

The first condition represents a threshold dividing the saddle-type attractor of the toppling acceleration into stable and unstable manifolds. The stable manifold briefly occurs when the tilt angle is moving toward zero, while the unstable manifold is characterized by falling away from zero. The angle of the dividing line is given by the slope parameter *α*. The second condition describes a radius (*r*) about the origin within which the tilt angle is too small to be detected or too stable for immediate correction (note that *r* has conventionally been used to denote the delay time interval in the delay differential equation literature. Here we have preferred *τ* for that purpose.). By converting the switching threshold slope *α* into the angle *a* as $\alpha =\frac{\sin(a\pi )}{\cos(a\pi )}$, we change the upper and lower estimation bounds from [−∞, ∞] to [0, 1]. This way, the parameter *a* represents the percentage of the phase space, not including the insensitivity radius, for which the active control parameters are non-zero.

The estimable parameters of the SDDE are summarized in Table 1. Many of the parameters have previously been estimated in a variety of ways, sometimes with highly varied results. Tietäväinen et al. [18] used the approximate Bayesian inference [17] with data simulation to estimate *P*, *D*, *a*, *τ*, and *σ*. Among these, the method failed to obtain precise distributions for *D* in both simulations and empirical application. It is also not clear whether fixing the other parameters to uncertain theoretical priors (*K* = .8, *B* = 4, and *r* = .004) results in biased estimates. Direct physiological measurements found the relative resistance to toppling torque at the ankle, *K*, to be as high as 91% on average [22] when the average magnitude of disturbance is small. Another study estimated relative resistance to be around 64% when disturbances were larger [23]. Conversely, the chosen value of *r* involves a conjecture about perceptual sensitivity that is specific to this model and has not been measured directly.

**Table 1:** Parameters of the IPC model with units and descriptions.

Fixed / Observed		Unit
*I*	Inertia	(kgm)^2^
*m*	Body mass	(kg)
*h*	Distance of center of mass from the ankle	(m)
*g*	Acceleration from gravity	(m/s^2^)
Estimated		
*K*	Intrinsic upright stiffness	% (of total Nm/rad)
*B*	Joint friction	Nms/rad
*P*	Active response force	Nm/rad
*D*	Active response damping	Nms/rad
*a*	Percentage of phase space active	%
*r*	Insensitivity radius	rad
*τ*	Feedback delay	s
*σ*	Process noise variance	Nm
*ϵ*	Measurement error variance	rad

Tietäväinen et al. [18] obtained a value of *τ* around 300 ms, while other methods of assessment have produced estimates including 125 ms [24] and 200 ms [21]. Direct measurements of ankle response, however, found response to start at 30 ms with maximal displacement around 120 ms [25]. If feedback delay is too long, then intermittent periods of acceleration due to gravity or muscle feedback will be consequently prolonged even as the state enters unstable regions of the phase space. One result is overcompensation for error, in which the fast oscillations found in sway are more amplified than would be the case with shorter delays. Alternatively, if the value of *a* is too high, then delayed feedback may bypass the unstable manifold and activate at inappropriate locations in the phase space, potentially amplifying slower oscillations over time. Long feedback delays can therefore contribute to instability, sway amplification, and higher risk of falling, but the exact kinds of error are determined by the joint behavior of several parameters, including *a*, *r*, and disturbance magnitude *σ* [1].

### Estimation

The above equations represent a Stochastic Delay Differential Equation (SDDE). The Kalman-Bucy filter provides minimum-variance unbiased estimates of the state of a stochastic process when both measurement and process noise are present and can be used to estimate the parameters of continuous-time differential equations from noisy data [26]. However, two challenges arise when estimating the parameters of an SDDE, including the lag interval *τ* and the lagged position and velocity coefficients, *P* and *D*. First, interpolation of the lagged states must be used to allow a continuous domain of possible values for *τ*. Second, backward extrapolation must be used to estimate the unmeasured interval of lagged states preceding initial state **x**_0_.

Last, we address problems that occur when the discrete switching conditions are toggled between measured instances. For most intervals between measures, the dynamics are linear and the prediction is exact, but state predictions that traverse the condition thresholds will systematically introduce bias to the linear dynamics unless the correct ratio of active and inactive dynamics within each traversal is estimated. We detail an algorithm to resolve this bias by adjusting the prediction according to each of the possible threshold-traversal scenarios.

#### Optimal filtering

The state-space equation for the time-lagged IPC system is given as

$$\begin{array}{cc} {\dot{x}}_{t}= & Ax_{t}+A_{\tau }x_{t-\tau }+Q, \end{array}$$ (5)

$$\begin{array}{cc} y_{t}= & Hx_{t}+\mu +R, \end{array}$$ (6)

where Q is the process noise covariance matrix, H is the measurement matrix, *μ* is the estimated origin about which the COM oscillates, and R is the covariance matrix of measurement error. The contemporaneous and lagged state vectors are

$$\begin{array}{cc} x_{t}= & [\begin{array}{c} x_{t}\\ {\dot{x}}_{t} \end{array}],\;{\dot{x}}_{t}=[\begin{array}{c} {\dot{x}}_{t}\\ {\ddot{x}}_{t} \end{array}],\;x_{t-\tau }=[\begin{array}{c} x_{t-\tau }\\ {\dot{x}}_{t-\tau } \end{array}], \end{array}$$

and the state transition matrices are

$$\begin{array}{cc} A= & [\begin{array}{cc} 0 & 1\\ mgh(1-K)/I & -B/I \end{array}],\;A_{\tau }=[\begin{array}{cc} 0 & 0\\ -mghP/I & -D/I \end{array}],\;Q=[\begin{array}{cc} 0 & 0\\ 0 & \sigma^{2} \end{array}], \end{array}$$

Matrix A contains the parameters of the passive, instantaneous forces, while A_*τ*_ contains the conditional parameters of active feedback. When the conditions given in Eq 3 evaluate to false, A_*τ*_ = 0.

The measurement matrices simply attribute the observed COM to the state position with estimated origin *μ* and measurement error variance *ϵ*:

$$\begin{array}{cc} H= & [\begin{array}{cc} 1 & 0 \end{array}]\;R=[\begin{array}{c} \epsilon \end{array}]\; \end{array}$$

The complete algebra for the prediction and correction steps of Kalman Filtering is excluded, as its derivation can be found in many resources [27] and remains largely unchanged for this model. However, the key difference in this case is that the prediction step is altered to include the delayed term. Using the following matrix discretizations,

$$\begin{array}{cc} A^{d}= & e^{A\Delta t}, \end{array}$$ (7)

$$\begin{array}{cc} A_{\tau }^{d}= & A^{-1}\left(A^{d}-I\right)A_{\tau } \end{array}$$ (8)

$$\begin{array}{cc} Q^{d}= & \int_{\delta =0}^{\Delta t}e^{A\delta }Qe^{A\delta \;\text{T}}d\delta \end{array}$$ (9)

we can then provide the prediction equations for the state mean and covariance as follows:

$$\begin{array}{cc} {\hat{x}}_{t+\Delta t}= & A^{d}{\bar{x}}_{t}+A_{\tau }^{d}{\bar{x}}_{t-\tau +\Delta t} \end{array}$$ (10)

$$\begin{array}{cc} {\hat{P}}_{t+\Delta t}= & A^{d}{\bar{P}}_{t}A^{d\;\text{T}}+A_{\tau }^{d}{\bar{P}}_{t}A_{\tau }^{d\;\text{T}}+Q^{d} \end{array}$$ (11)

For stationary series with large number of observations, **P**_*t*_ ≈ **P**_∞_. For convenience, we use **P**_*t*−1_ as an approximation to **P**_*t*−*τ*_. Note that Eq 8 does not work if *K* = 1, making **A** singular. However, small, numerically viable deviations from *K* = 1 will not substantially impact solution topology. Point singularitieswill also not impede derivative-free optimization methods.

#### Estimation of feedback delay

**Linear interpolation** To obtain estimates of the state at time lags that do not fall on measurement instances, we use linear interpolation of the state:

$$\begin{array}{cc} \lambda = & \frac{\tau }{\Delta t}, \end{array}$$ (12)

$$\begin{array}{cc} {\hat{x}}_{t-\tau }= & x_{i-\left\lfloor \lambda \right\rfloor }+\left(x_{i-\left\lfloor \lambda \right\rfloor }-x_{i-\left\lceil \lambda \right\rceil }\right)\left(\lambda -\left\lfloor \lambda \right\rfloor \right), \end{array}$$ (13)

λ is the conversion of the time delay to the number of measured occasions comprising that interval. The ceiling and floor functions thus give valid measurement indices and are used to give a combination of measurements falling to either side of λ, weighted proportionally. If *τ* = 0, then the second term of Eq 13 can be neglected.

**Backward extrapolation of initial values** By introducing an initial value parameter for acceleration, we can estimate a quadratic extrapolation backward from *t*_0_ to *t*_0_ − *τ*, allowing the influence of lagged states and switching conditions to be respected within the first λ iterations of filtering:

$$\begin{array}{c} \text{If}\;t\leq \tau \{\begin{array}{cc} {\hat{x}}_{t-\tau }= & x_{0}+{\dot{x}}_{0}\left(t-\tau \right)+{\ddot{x}}_{0}{\left(t-\tau \right)}^{2},\\ {\hat{\dot{x}}}_{t-\tau }= & {\dot{x}}_{0}+2{\ddot{x}}_{0}\left(t-\tau \right), \end{array} \end{array}$$ (14)

#### Constrained interpolation of dynamic switching points

To avoid bias due to missing transitional information between measures that straddle the threshold of the conditions given by Eq 3, we explicitly detect each case, interpolate the state falling on the condition threshold, and predict its traversal in two steps. For convenience, take the shortened terms *u* and *v* as the delayed states leading up to, and away from the condition threshold:

$$\begin{array}{c} u≔x_{t-\tau },\;\dot{u}≔s{\dot{x}}_{t-\tau }\\ v≔x_{t-\tau +\Delta t},\;\dot{v}≔s{\dot{x}}_{t-\tau +\Delta t}, \end{array}$$ (15)

Where *s* is the seconds constant, such that $v,u,\dot{v}$, and $\dot{u}$ are measured in radians. For use later, we note here that the slope between the two points is $m=\frac{\dot{v}-\dot{u}}{v-u}$.

**Conditions for switching off**:

$$\begin{array}{c} \text{If}\;\left[u\left(\dot{u}-\alpha u\right)>0\;\text{and}\;u^{2}+{\dot{u}}^{2}>r^{2}\right]\;\text{and}\;\left[v\left(\dot{v}-\alpha v\right)\leq 0\;\text{or}\;v^{2}+\dot{v^{2}}\leq r^{2}\right], \end{array}$$ (16)

then A_*τ*_ is switching off. If this holds true, then the following conditions further apply:

$$\begin{array}{c} \text{If}\;v^{2}+{\dot{v}}^{2}>r^{2}\\ \text{and}\;![\left(v>0\;\text{and}\;\dot{v}<0\;\text{and}\;u<0\;\text{and}\;\dot{u}<0\right)\\ \text{or}\;(v<0\;\text{and}\;\dot{v}>0\;\text{and}\;u>0\;\text{and}\;\dot{u}>0)], \end{array}$$ (17)

then the lagged state is traversing the line $\dot{x}=\alpha x$ outside of the slack radius and not traversing *u* = 0. The interpolated point $\left(\hat{u},\hat{\dot{u}}\right)$ falls on the line, and is calculated as

$$\begin{array}{c} \hat{u}=-\frac{mv-\dot{v}}{\alpha -m},\;\hat{\dot{u}}=m\hat{u}-mv+\dot{v}, \end{array}$$ (18)

If $v^{2}+{\dot{v}}^{2}\leq r^{2}$, then the lagged state is traversing into the slack radius, and the interpolated point is

$$\begin{array}{c} \hat{u}=\frac{r^{2}+v^{2}+2mv\dot{v}-{\dot{v}}^{2}}{2(v+m\dot{v})},\;\hat{\dot{u}}=m\hat{u}-mv+\dot{v}, \end{array}$$ (19)

In all other cases in which (16) holds true, *u* is traversing the axis at *u* = 0.

$$\begin{array}{c} \hat{u}=0,\;\hat{\dot{u}}=-mv+\dot{v}, \end{array}$$ (20)

**Conditions for switching on**: For cases where the delayed feedback is switching on, the roles of *u* and *v* are simply traded. The interpolated point is calculated identically under each set of conditions analogous to those for switching off.

$$\begin{array}{c} \text{If}\;\left[u\left(\dot{u}-\alpha u\right)\leq 0\;\text{or}\;u^{2}+{\dot{u}}^{2}\leq r^{2}\right]\;\text{and}\;\left[v\left(\dot{v}-\alpha v\right)>0\;\text{and}\;v^{2}+\dot{v^{2}}>r^{2}\right], \end{array}$$ (21)

then **A**_*τ*_ is switching on. If this holds true, then the following conditions further apply:

$$\begin{array}{c} \text{If}\;v^{2}+{\dot{v}}^{2}<r^{2}\\ \text{and}\;![\left(u>0\;\text{and}\;\dot{u}<0\;\text{and}\;v<0\;\text{and}\;\dot{v}<0\right)\\ \text{or}\;(u<0\;\text{and}\;\dot{u}>0\;\text{and}\;v>0\;\text{and}\;\dot{v}>0)], \end{array}$$ (22)

then the lagged state is traversing the line $\dot{x}=\alpha x$ outside of the slack radius and not traversing *u* = 0, and the interpolated point is calculated as Eq (18). If $u^{2}+{\dot{u}}^{2}\leq r^{2}$, then the lagged state is traversing the slack radius from within, and the interpolated point is calculated with Eq (19). In all other cases in which Eq (21) holds true, *u* is traversing the axis at *u* = 0 and the interpolated point is calculated as Eq (20).

**Prediction for threshold traversal**: The time for *u* to reach the switching threshold, Δ*t*^−^, and the time to reach the next observation after the threshold, Δ*t*^+^, can be calculated from the interpolated state at the threshold and its neighboring states, *u* and *v*:

$$\begin{array}{c} \Delta t^{-}=\frac{∥u-\hat{u}∥}{∥v-u∥}\Delta t,\;\Delta t^{+}=\frac{∥v-\hat{u}∥}{∥v-u∥}\Delta t, \end{array}$$ (23)

In the first step, **A**, **A**_**τ**_, and **Q** are discretized for the interval Δ*t*^−^, and the prediction is given as:

$$\begin{array}{cc} {\hat{x}}_{t+\Delta t^{-}}= & A^{d}{\bar{x}}_{t}+A_{\tau }^{d}u \end{array}$$ (24)

$$\begin{array}{cc} {\hat{P}}_{t+\Delta t^{-}}= & A^{d}{\bar{P}}_{t}A^{d\;\text{T}}+A_{\tau }^{d}P_{t}A_{\tau }^{d\;\text{T}}+Q^{d}. \end{array}$$ (25)

In the second step, **A**, **A**_**τ**_, and **Q** are discretized for the interval Δ*t*^+^, and the prediction is computed from time *t* + Δ*t*^−^ as:

$$\begin{array}{cc} {\hat{x}}_{t+\Delta t}= & A^{d}{\hat{x}}_{t+\Delta t^{-}}+A_{\tau }^{d}\hat{u} \end{array}$$ (26)

$$\begin{array}{cc} {\hat{P}}_{t+\Delta t}= & A^{d}{\hat{P}}_{t+\Delta t^{-}}A^{d\;\text{T}}+A_{\tau }^{d}{\hat{P}}_{t+\Delta t^{-}}A_{\tau }^{d\;\text{T}}+Q^{d}. \end{array}$$ (27)

For either step, $A_{\tau }^{d}=0$, depending on whether the active feedback is switching on or off.

#### Optimization

The toggling of active feedback is not a smooth process and results in discontinuities in the space of a cost function for fitting the model, though these are greatly mitigated by the interpolation measures described above. The complexity of the model nonetheless gives rise to multiple local solutions, and attempts to find optimal parameters using local methods such as gradient descent and Nelder-Mead reliably fail. Instead, we recommend using a method of global, derivative-free optimization such as Differential Evolution (DE) [28]. The optimization parameters that we chose are listed below.

- Strategy: DE / rand / 1 / bin with per-vector-dither
- Iterations = 15000
- Population size = 30
- Crossover Probability (CR) = .95
- F = .15
- Weighting of successful members (c) = 0
- Step tolerance: 500
- Relative tolerance: 1e-10

We chose a high crossover probability (CR) due to high dependence between parameters of the model and used simulations to confirm reasonable convergence given the chosen population size, iterations, and F value. DE does not require initial values for parameter estimation, but instead populates a region within explicit bounds. The bounds used here for simulation and data analysis are given in Table 2. Parameter bounds were generally restricted to potentially stable and theoretically meaningful ranges, such as for *K*, *P*, *r*, and *τ*. Theoretical interpretations of parameters *B* and *D* were less certain and were therefore allowed to vary beyond boundaries imposed under any particular physiological definition. *τ* was constrained to the extremes of the empirical distribution of neural delay given the results from Peterka [21]. Otherwise, bounds were made extreme enough to capture all reasonable possibilities without unnecessarily slowing convergence.

**Table 2:** Optimization bounds for all parameters.

Par.	Domain
*K*	[0, 1]
*B*	[-1000, 1000]
*P*	[0, 2]
*D*	[-1000, 1000]
*a*	[0, 1]
*r*	[0, 2]
*τ*	[0.15, 0.25]
*σ*_*w*_	[0, 5]
*σ*_*ϵ*_	[0, 1]
*x*_0,*i*_	[-10, 10]
${\dot{x}}_{0,i}$	[-50, 50]
${\ddot{x}}_{0,i}$	[-100, 100]
*μ*_*i*_	[-20, 20]

#### Software

All analyses used R statistical programming environment [29]. Differential Evolution was provided by the R package DEoptim [30]. The IPC model was implemented in C++ using R packages Rcpp [31] and RcppArmadillo [32], and compiled to the open-source R package IPCmodel. The package includes the following functions:

- ipcModel(): C++ Kalman Filter with delayed terms and switching conditions that returns a -2Log-likelihood value for optimization.
- ipcSimulate(): C++ numerical integrator that generates simulated data for the IPC model.
- ipcMultiGroup(): R wrapper for ipcModel() that incorporates physical constants, parameter algebras, and enables the estimation of both within and between-series parameters.
- kalmanIntegrate(): C++ helper function that accepts continuous-time state-space matrices and returns discretized matrices for Kalman-Bucy filtering.

## Simulations

Two simulations were used: the first to check model specification, and the second to evaluate the accuracy and precision of parameter estimates. The first simulation used noiseless (i.e. deterministic trajectories) with perfect measurement to check for systematic bias due to the estimation strategy. The second simulation used data simulated to include both process and measurement noise according to the possible properties of real data recorded by a force plate. Solutions for both deterministic and noiseless simulations were generated in linearized steps of size 10^−5^s then downsampled according to the design of each simulation. This procedure ensured both numerical accuracy of solutions and simulated real world mapping of analogue processes to discrete measurements. The statistical properties of simulated series were expected to be invariant to downsampling due to the fractal property of continuous random walks (i.e., Wiener processes) where Δ*t* ∼ *N*(0, Δ*t*).

### Parameter sets

Six sets of simulated parameters were defined to test the model’s estimation capability over a variety of possible behaviors and are shown in Table 3. The first set replicates the simulated data for Model 4 by Asai et al. [1] and is named accordingly. The second and third sets (“Low Noise” and “High Noise”) respectively decrease and increase the variance of process noise to examine its effect on other parameters. The fourth and fifth(“Active Control” and “Passive Control”) sets respectively increase and decrease the ratio of active to passive control, representing different plausible configurations for stability. The sixth set (“Rambling and Trembling”) represents a stationary random-walk series that diverges markedly from the underlying theory but is nonetheless a stable and plausible configuration.

**Table 3:** Parameter sets for generating simulated data.

	*K*	*B*	*P*	*D*	*a*	*r*	*τ*	*σ*	*ϵ*
Asai et al.	0.80	4.00	0.25	10.00	0.62	0.40	0.20	0.20	1E-04
Low Noise	0.80	4.00	0.25	10.00	0.62	0.40	0.20	0.05	1E-04
High Noise	0.80	4.00	0.25	10.00	0.62	0.40	0.20	1.00	1E-04
Passive Control	0.95	4.00	0.15	10.00	0.50	0.70	0.20	0.20	1E-04
Active Control	0.75	4.00	0.70	120.00	0.80	0.20	0.20	0.20	1E-04
Rambling and Trembling	0.98	500.00	0.20	-50.00	0.45	0.05	0.20	2.00	1E-04

### Sim 1: Noiseless series

**Fig 1:**
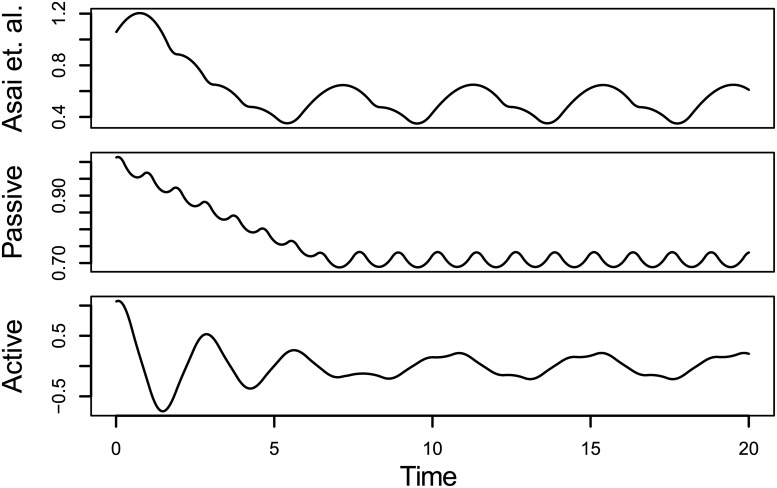
Noiseless series with initial values $x_{0}=1,{\dot{x}}_{0}=0$.

To test for improper model specification and systematic sources of bias, noiseless series were generated to span 20s, with a step size of 10^−5^s, then downsampled to an observation every 0.01s and again to every 0.1s. The noiseless series used in the first simulation are shown in Fig 1. Only one series per set and per downsample rate was used, as there were no sources of sampling error. To ensure that estimates converged to a high degree of precision, 3000 iterations of optimization were used.

Table 4 contains the parameter estimates for these simulations, with sampling rate shown to the left. Only the velocity coefficients *B* and *D* exhibited substantial bias all throughout, and the “High Active” set incurred the greatest bias over nearly all parameters. Most parameter estimates given 100Hz sampling were exact to at least 3-5 decimal places. Reducing sampling resolution by a factor of ten increased biases to parameters *B* and *D* by a factor of ten to fifteen, but much less so for *K* and *P*. The nonlinear parameters *a*, *r*, and *τ* exhibited the least bias for all sets.

**Table 4:** Parameter point estimates for simulated noiseless series. Downsampling rate in Hz is shown in the left column. See Table 3 for the true, data-generating parameter values of each set.

Hz	Set	*K*	*B*	*P*	*D*	*a*	*r*	*τ*	*σ*
100	Asai et al.	0.80003	4.10640	0.24996	10.62579	0.62000	0.40000	0.20001	0.00000
	Passive Control	0.95000	4.08142	0.14998	10.46709	0.50000	0.70000	0.20000	0.00000
	Active Control	0.75086	4.09833	0.69372	122.07313	0.79987	0.19990	0.20020	0.00000
10	Asai et al.	0.80024	5.65678	0.24975	15.62635	0.62030	0.39914	0.20000	0.00000
	Passive Control	0.95001	5.40384	0.14939	19.03988	0.49999	0.70016	0.19978	0.00001
	Active Control	0.74853	4.17357	0.63636	140.82292	0.80120	0.19951	0.20097	0.00022

The small biases to *K*, *B*, *P*, and *D* most likely occur as a result of the approximate, linear interpolation methods and inability to account for process noise before *t*_0_ in the quadratic backward extrapolation. Biases may be further mitigated using polynomial interpolation of the lagged state. However, the exact accuracy of the estimated *τ* indicates that bias from linear interpolation is probably trivial in this case.

A second source of bias may be the limits of numerical precision. When no noise is present in the system, the state only occupies a small area of the phase space where certain values of *B* and *D* may have nearly unobservable effects on the solution. We show later that relatively unbiased estimates of *B* and *D* can indeed be obtained as a function of the other parameters, including the process noise variance *σ*.

### Sim 2 Estimation from noisy data

**Fig 2:**
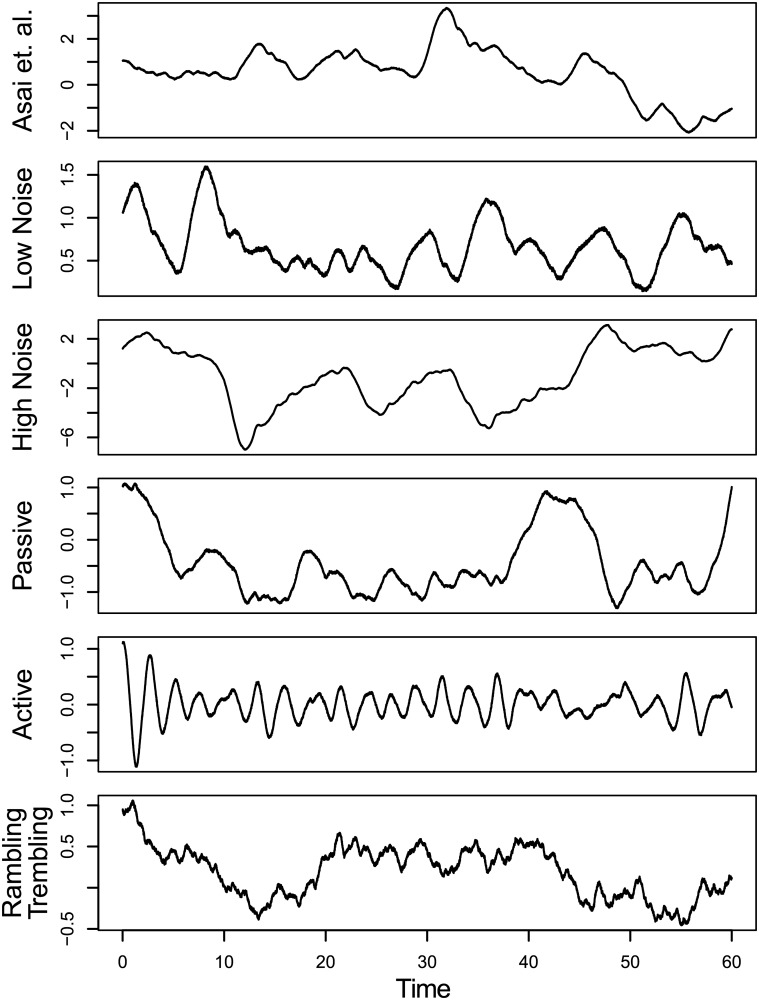
Examples of simulated series from six parameter sets.

To test the precision and accuracy of IPC parameter estimates given the dimensions and expected structure of the data from Santos et al. [20], one-hundred individuals were simulated for each parameter set in Table 3, with examples series shown in Fig 2. Each individual consisted of three trials, and each trial consisted of a 60s series downsampled to 100 Hz. The same parameters were estimated for all three trials, making for a total of 18,000 observations per individual model.

**Fig 3:**
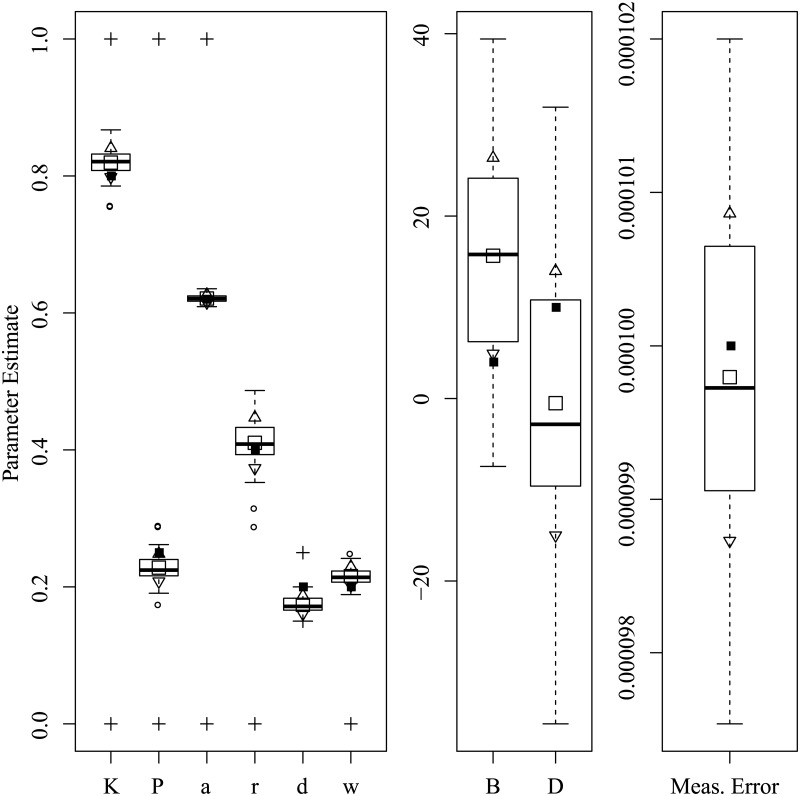
Parameter recovery results for the Asai et al. replication set. Black squares: data-generating value; Empty squares: Estimate mean; Triangles: Upper and lower std. dev.; Circles: Outliers; Crosses: Optimization boundaries.

Figs 3 through 8 show the sampling variation and bias for each parameter set. Boxplots are grouped by common axis scale. Table 5 gives the means and standard deviations of each parameter for each set.

**Fig 4:**
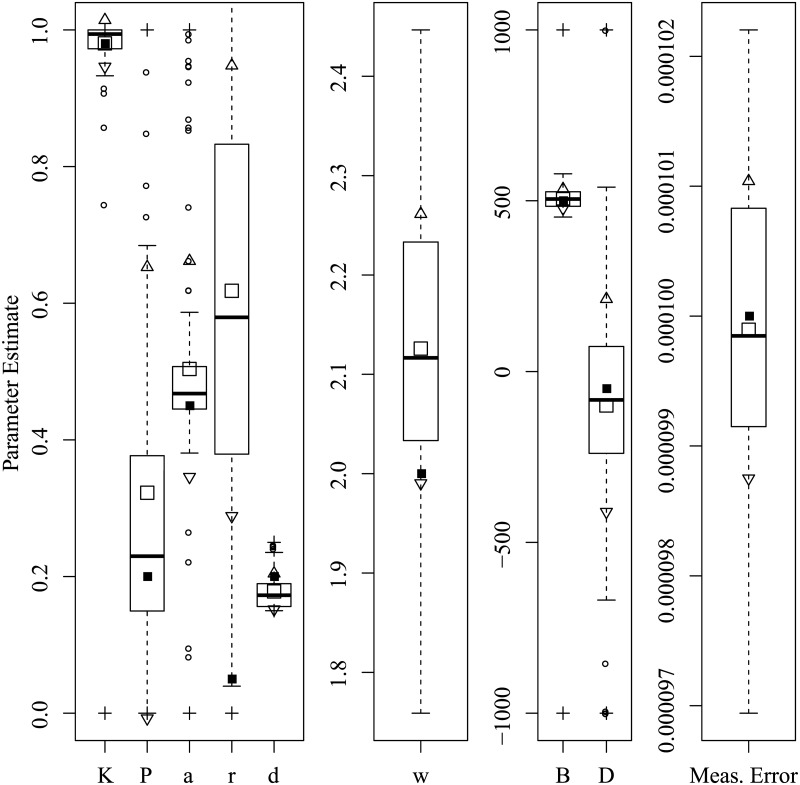
Parameter recovery results for the Rambling and Trembling set.

**Fig 5:**
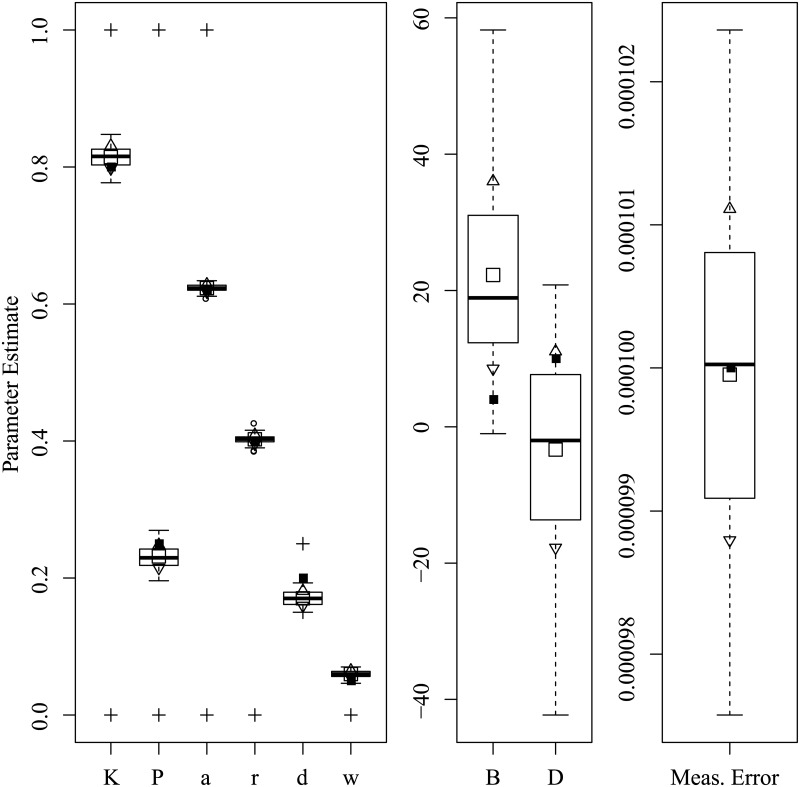
Parameter recovery results for the Low Noise set.

**Fig 6:**
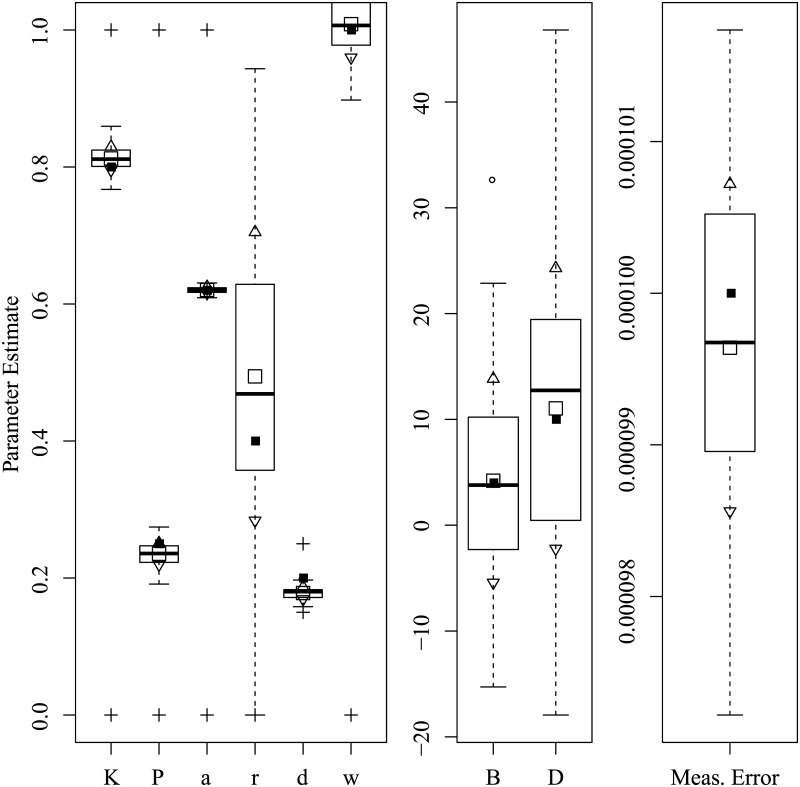
Parameter recovery results for the High Noise set.

**Fig 7:**
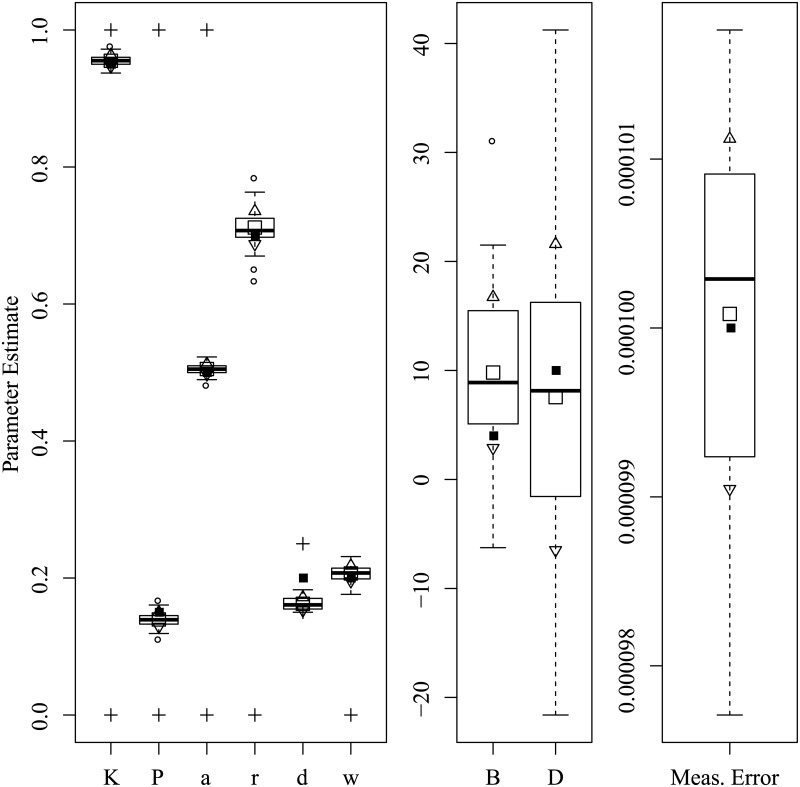
Parameter recovery results for the Passive Control set.

**Fig 8:**
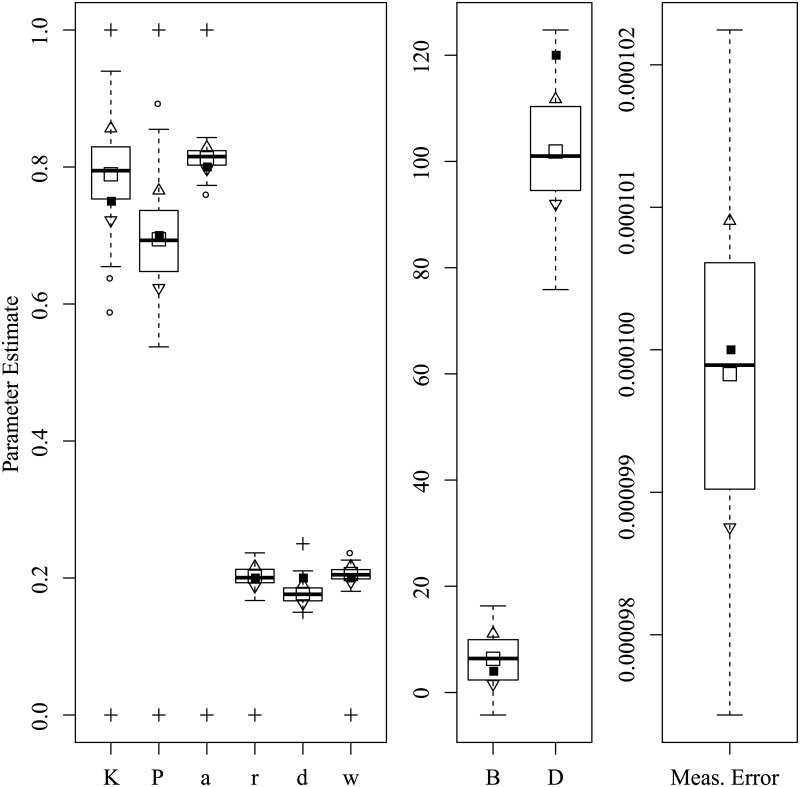
Parameter recovery results for the Active Control set.

**Table 5:** Simulation results: Means (*μ*) and standard deviations (*σ*) of estimated parameters over 100 iterations of simulation for six parameter sets. True values are given in Table 3, and parameter descriptions are given in Table 1.

Par.		Asai et al.	Low Noise	High Noise	Low Active	High Active	Ramb./Tremb.
*K*	*μ*	0.820	0.814	0.812	0.955	0.789	0.978
	*σ*	0.021	0.017	0.017	0.008	0.067	0.025
*B*	*μ*	15.654	22.278	4.203	9.811	6.350	504.441
	*σ*	10.727	13.743	9.631	6.922	4.743	28.426
*P*	*μ*	0.228	0.231	0.235	0.139	0.694	0.301
	*σ*	0.020	0.017	0.016	0.011	0.071	0.211
*D*	*μ*	-0.511	-3.337	11.039	7.556	101.869	-138.941
	*σ*	14.490	14.398	13.258	14.042	9.850	281.247
*a*	*μ*	0.621	0.623	0.620	0.505	0.813	0.481
	*σ*	0.006	0.005	0.004	0.008	0.016	0.072
*r*	*μ*	0.410	0.402	0.494	0.712	0.202	0.609
	*σ*	0.037	0.006	0.210	0.024	0.014	0.322
*τ*	*μ*	0.174	0.170	0.178	0.162	0.177	0.176
	*σ*	0.013	0.011	0.009	0.010	0.013	0.023
*σ*	*μ*	0.216	0.060	1.008	0.207	0.206	2.117
	*σ*	0.014	0.005	0.049	0.011	0.011	0.134
*ϵ*	*μ*	9.98E-05	1.00E-04	9.96E-05	1.00E-04	9.98E-05	9.99E-05
	*σ*	1.07E-06	1.16E-06	1.08E-06	1.04E-06	1.08E-06	1.15E-06

Variance and baises across all parameters were highly interdependent. Estimates of both process noise (*σ*) and measurement error were precise and close to their true values, indicating successful filtering of the state. The precision of active and passive and active control parameters depended on the true values of parameters and resulting behavior of the process. For the Asai et al. replication and the sets with low and high process noise, most control parameters had only small bias and high precision, while others were less reliable under particular conditions. The greatest apparent contrast may be the insensitivity radius *r*, which was not estimable for the Rambling and Trembling set in which its true value was small, and much less reliable in the increased noise set where its value matched Asai et al.

The *B* and *D* parameters were the least reliable, and are possibly empirically unidentified without a sufficiently high process noise variance. This is evident from the increased noise set (Fig 6) and the Rambling and Trembling set (Fig 4). The active control set (Fig 8) also showed successful estimation of the *B* parameter, and improvements in estimating *D* over the the Asai et al. set, low noise, and passive control.

From the variation in results across sets, it can be inferred that a parameter can only be estimated reliably when the state occurs for a sufficient amount of time in the portions of the phase space for which that parameter has an influence. For instance, the insensitivity radius will not be estimable if the state tends to bypass it entirely. This may be due to a large variance of process noise, or for large values of *B* that distort the saddle shape of the passive attractor space, causing an orbital path that never intersects the origin. Likewise *B* and *D* cannot be estimated reliably if the process does not frequently visit the extremes of the phase space where their influence is most apparent.

Empirical under-identification of some parameters is not necessarily problematic for the others, and does not imply the unreliable parameters should be fixed to some value or excluded. Two solutions to empirical under-identification are to increase the length and resolution of the sample to increase the chances of observing informative behavior, and perhaps to introduce small interventions or disturbances such that subjects express the full range of relevant dynamic behaviors.

Standing apart from the other parameters is the feedback delay *τ*. Despite perfect accuracy in the noiseless case, it tended to bias downwards when estimated from noisy data. It is unclear from our simulations why the bias occurs and whether it accounts for bias to other parameters. However, the estimates were not generally boundary cases, and the sampling variability was small. If the bias is consistent, the delay parameter should still be comparable between persons, with the caveat that the estimate is understated by 20-40ms.

## Data analysis

The IPC model was fit to empirical postural control data to 1) estimate the multivariate distributions of each parameter, 2) test for expected effects from age and visual feedback, 3) test the consistency of parameters within-person, 4) compare the proposed model to simpler alternatives. COM data were obtained from the data set published for public use by Santos et al. [20] and included 49 individuals at 100Hz for 60 seconds per trial. Three trials were conducted with eyes open, and three with eyes closed. Only trials tested with a rigid floor were used for our analyses. Height and weight were provided for each individual and included as the constants *h* and *m* in the model, scaled to units of meters and kilograms respectively. Height was scaled by 0.51, the approximate ratio of vertical COM to total height in upright standing (calculated from Table 1, p.7 of [23]). By visual inspection of the sample, it was found that the first and last several seconds of many series contained large, sudden changes in position likely relating to movement during the initiation and termination of the trial period. To ensure that only the stationary dynamics of interest were modeled, 500 occasions were trimmed from the beginning and end of each series, leaving 5000 occasions or 50 seconds of data per trial, and 30,000 measurements in total per individual.

### Models

Three models were fit to each of three trials per individual to examine the statistical significance of the parameters involved in intermittent activation and delayed feedback. The models included, in descending order of complexity, the complete intermittent stochastic delay differential equation (ISDDE),

$$\begin{array}{c} I{\ddot{\theta }}_{t}=mgh\left(1-K\right)\theta_{t}+B\dot{\theta_{t}}+mghf_{P}\left(\theta_{t-\tau }\right)+f_{D}\left({\dot{\theta }}_{t-\tau }\right)+\sigma w_{t}, \end{array}$$ (28)

a stochastic delay differential equation (SDDE) with delayed feedback but no intermittent switching conditions,

$$\begin{array}{c} I{\ddot{\theta }}_{t}=mgh\left(1-K\right)\theta_{t}+B\dot{\theta_{t}}+mghP\theta_{t-\tau }+D{\dot{\theta }}_{t-\tau }+\sigma w_{t}, \end{array}$$ (29)

and a stochastic differential equation (SDE) containing only instantaneous, continuous PID control terms:

$$\begin{array}{c} I{\ddot{\theta }}_{t}=mgh\left(1-K\right)\theta_{t}+B\dot{\theta_{t}}+\sigma w_{t}. \end{array}$$ (30)

All models included trial-specific initial conditions *x*_0,*i*_ and ${\dot{x}}_{0,i}$ and sway origins *μ*_*i*_ for *i* ∈ [1, 2, 3]. The ISDDE and SDDE both included trial-specific estimation of ${\ddot{x}}_{0,i}$ for backward extrapolation. All models included measurement error variance *σ*_*ϵ*_. Parameter boundaries, shown in Table 6 reflected both theoretical and analytic roles of each parameter. For example, *B* could not be less than zero in the ISDDE because it is conjectured to represent ankle stiffness, and stability is required to come from values of *P* and *D* in the given domains. In the SDE, stable solutions must rely on only instantaneous feedback with coefficients *K* and *B*. In the absence of other theoretical mechanisms, the same physiological interpretations of *K* and *B* could not be assumed and thus the same theoretical constraints were not applied.

**Table 6:** Optimization boundaries [lower, upper] for each parameter, under each model.

Par.	ISDDE	SDDE	SDE
*K*	[0, 1]	[0, 1]	[-10, 10]
*B*	[0, 2000]	[0, 2000]	[-2000, 2000]
*P*	[0, 2]	[-2, 2]	
*D*	[-2000, 2000]	[-2000, 2000]	
*a*	[0, 1]		
*r*	[0, .1]		
*τ*	[0, 1]	[0, 1]	
*σ*	[0, 5]	[0, 5]	[0, 100]
*ϵ*	[0, .03]	[0, .03]	[0, 10]
*x*_0_	[-5, 5]	[-5, 5]	[-5, 5]
${\dot{x}}_{0}$	[-150, 150]	[-150, 150]	[-150, 150]
${\ddot{x}}_{0}$	[-500, 500]	[-500, 500]	
Origin	[-1, 1]	[-1, 1]	[-1, 1]

Multiple regression was used to test the association between each parameter, visual feedback, and age, accounting for height and mass as covariates. Pearson correlation was used to estimate the correlation between parameter estimates during trials with eyes open and trials with eyes closed. Maximum likelihood estimation was used to fit each model, assuming the multivariate normality of measurement and process noise.

The estimated means $\hat{\mu }$, standard deviations $\hat{\sigma }$, and medians of each estimated parameter across all trials × participants × visual feedback conditions, are given in Table 7. The estimated individual-level intraclass correlations (*ρ*_*ICC*_), effect sizes, and *p*-values for age and visual feedback are also given for each model. Measurement error estimates were generally small (*σ*_*ϵ*_ < 1*e* − 3) and were omitted from the tables. Minor, trial-specific “nuisance” parameters including sway origins and initial values were also omitted. Mean sway origin was estimated to be 0.217, with a standard deviation of 0.11 and a median of 0.226.

**Table 7:** Summary statistics, effects of age and vision accounting for height and mass, and person-level intraclass correlations of parameter estimates under each model. Bonferroni adjusted *α* = .0029.

		$\hat{\mu }$	$\hat{\sigma }$ (Trimmed)	Median	*β*_*Vision*_	CI	*p*	*β*_*Age*_	CI	*p*	*ρ*_*ICC*_
ISDDE	*K*	0.920	0.021 (0.019)	0.920	0.002	(0, 0.004)	0.133	7.6e-5	(1.9e-5, 1.3e-4)	*0.009	0.912
	*B*	3.057	10.223 (5.2)	0.000	1.019	(-1.314, 3.352)	0.393	-0.078	(-0.144, -0.012)	*0.023	0.191
	*P*	0.174	0.272 (0.066)	0.116	0.048	(-0.015, 0.111)	0.132	-0.002	(-0.004, 0)	0.074	-0.025
	*D*	154.789	160.153 (47)	131.123	-20.307	(-57.37, 16.76)	0.284	-0.043	(-1.097, 1.011)	0.936	0.040
	*a*	0.730	0.259 (0.26)	0.853	-0.055	(-0.114, 0.004)	0.069	0.001	(-0.001, 0.003)	0.275	0.049
	*r*	0.002	0.003 (0.0017)	0.002	0	(-0.001, 0.001)	0.572	0	(-2.2e-5, 2.2e-5)	0.971	-0.112
	*τ*	0.302	0.124 (0.095)	0.284	0.018	(-0.01, 0.046)	0.212	-0.001	(-0.002, 0)	*0.049	0.291
	*σ*_*w*_	0.047	0.006 (4.2e-4)	0.046	-0.001	(-0.002, 0)	0.086	4e-06	(0, 8e-6)	*0.033	0.490
SDDE	*K*	0.918	0.019	0.919	0	(-0.001, 0.001)	0.923	8.3e-5	(4.6e-5, 1.2e-4)	**1e-5	0.999
	*B*	0.838	3.508	0.000	0.163	(-0.647, 0.973)	0.693	-0.006	(-0.029, 0.017)	0.582	0.233
	*P*	0.162	0.071	0.146	-0.012	(-0.026, 0.002)	0.071	0.001	(0.001, 0.001)	**0.002	0.580
	*D*	68.818	53.280	50.384	-3.668	(-15.80, 8.47)	0.554	0.135	(-0.21, 0.48)	0.442	0.386
	*τ*	0.478	0.031	0.494	0.006	(-0.001, 0.013)	0.099	-2.4e-4	(-4e-4, -5e-5)	*0.015	0.034
	*σ*_*w*_	0.047	0.006	0.046	-0.001	(-0.002, 0)	*0.044	5e-6	(1e-6, 9e-6)	*0.012	0.494
SDE	*K*	0.931	0.020	0.930	0.004	(0.001, 0.007)	*0.007	1.8e-5	(-5.8e-5, 9.4e-5)	0.641	0.804
	*B*	17.475	13.231	14.616	2.462	(-0.475, 5.399)	0.102	-0.137	(-0.221, -0.053)	**0.001	0.608
	*σ*_*w*_	0.048	0.006	0.048	-0.002	(-0.003, -0.001)	*0.011	6e-6	(2e-6, 1e-5)	**0.002	0.544

* Significant at unadjusted *α* = .05

** Significant at adjusted *α* = .0029

**Fig 9:**
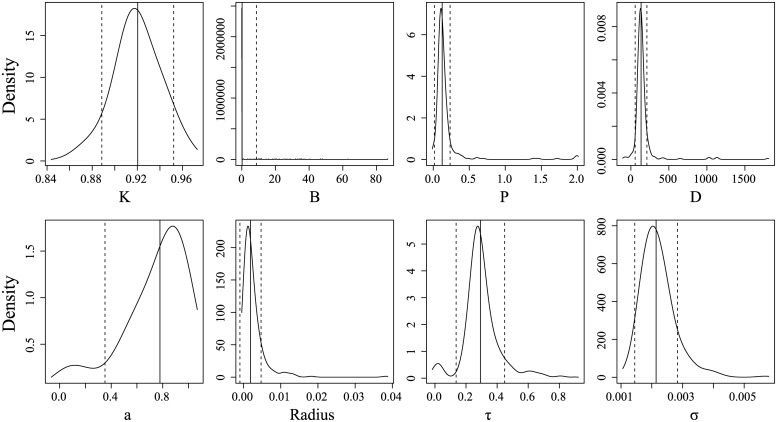
Marginal distributions of the ISDDE parameter estimates. Solid lines are means and dashed lines are standard deviations, both trimmed for the 15 highest values.

The estimated parameters of the ISDDE fell within the expected domains. Several parameters of the ISDDE had outliers that substantially inflated estimates of their standard deviations. Trimmed standard deviations in which the highest 15 values were excluded are given in parentheses in Table 7. The marginal distributions of each parameter with these trimmed means and standard deviations are shown in Fig 9. *B*, *P*, *D*, and *r* in particular were skewed upward by outliers but otherwise had relatively precise distributions about their medians, with similar precision to those of the SDDE. *K* had consistent values around .91 to .93 in all three models. *B* was close to zero for most series but skewed upward by outliers as high as 80. In the SDE, *B* was allowed to take negative values but had a mean around 17. All values of *B* in the SDE were positive and greater than zero, with a minimum of .82. *a* was generally high, representing active control over 75-85% of the phase space. Similarly, *r* was 50% smaller on average than values used in previous studies. *τ* had a median of 284 ms and was distributed between 200 to 400 ms. If the bias found in simulations is consistent and proportional, then the true median delay was closer to 240 ms. The SDDE estimated much longer delays on average at 470-490 ms but much lower values of *D*. Process noise standard deviation *σ*_*w*_ estimates were distributed identically between models.

No significant effects of visual feedback were observed in the parameters of any of the three models. The lowest *p*-values were for *a*(*p* = .069) and *σ*_*w*_(*p* = .086). Both the SDDE and SDE showed effects on *σ* with *p* < .05, the alpha level before adjusting for the 17 tests in total.

In the SDDE, both passive ankle stiffness *K* and active control coefficient *P* were shown to significantly increase with age. The effects were detected given the adjusted alpha level, with *p* < .0029. Both *B* and *σ*_*w*_ in the SDE showed significant trends with age as well. Other non-significant effects with *p* < .05 were *K* and *σ*_*w*_ in both the ISDDE and SDDE, *B* in the ISDDE, and *τ* in the SDDE. Effect estimates of *σ*_*w*_ were consistent across models.

Overall, parameters tended to be more consistent within person for the simpler models. The highest intra-class correlation for all parameters in all models was *K*, with extreme reliability (*ρ* = .999) in the SDDE. *σ* generally correlated around .5 for each model. The ISDDE had the least consistent parameters with intraclass correlations near zero for *P*, *D*, *a*, and *r*. The SDDE and SDE intraclass correlations were moderate to high for all except feedback delay, *τ*, which was near zero.

Akaike’s Information Criterion (AIC) [33], as $-2\text{ln}(\hat{L})+2k$ where *k* is the number of estimated parameters, was used to compare overall model fit for every individual. For each trial, the model with the lowest value of the AIC was selected as the best fitting option. In total, the ISDDE was selected for 227 trials, SDDE for 62, and SDE for 0. No significant associations were found between model selections over trials within person or by visual feedback condition.

## Discussion

### Simulation

Simulation studies were used to determine whether the parameters of the intermittent activation feedback control model proposed by Asai et al. [1] can be estimated using a Kalman Filtering-based framework with delayed proportional and derivative terms and discrete activation thresholds. The results of the simulations show that the parameters of the model can be estimated with relatively low bias and high precision if the behaviors for which they are influential are sufficiently expressed in the data (i.e., empirically identified). Every parameter of the model was successfully recovered in at least one of the parameter configurations tested, though no single configuration of parameters resulted in a completely unbiased set. The set of results shown in Fig 8 comes close, with downward bias only to the active derivative controller. We can also see by comparing Fig 6 to Figs 5 and 3 that an increased variance of process noise allowed the identification of the *B* and *D* parameters, but with large standard errors. The derivative coefficients were likely biased and unreliable when the trajectories did not frequent the extremes of position and velocity where the directional effects of derivative terms could be distinguished from other sources. Fig 4 shows that with process noise of a standard deviation much greater than the insensitivity radius and a weak attraction to point equilibria (*K* ≈ 1), the state is prone to drifting away from the origin where it will rarely traverse the insensitivity radius or switching boundary. If the data can be optimally explained without the use of the switching parameters, then they are said to be empirically unidentified. For this reason, both *a* and *r* do not contribute crucial information and converge to precise solutions when the data are optimally described by other parameter values characteristic of rambling and trembling. However, estimates of the derivative controllers *B* and *D* in that case were unbiased.

Across configurations, some iterations of model fitting resulted in negative values of *B*. In continual PID controllers, this would result in amplification and instability over time. In the ISDDE and SDDE, the stability of the system given a value of *B* depends on the corresponding values of *P*, *D*, and *a*, as the instantaneous proportional and derivative terms do not control the complete periodic behavior on their own. Negative values of *B* will promote further instability in the already unstable manifold of instantaneous feedback but will be counteracted when the state reaches the stable manifold determined by active feedback. It is informative that solutions occasionally involved negative values of *B* that breach its theoretical interpretation as joint friction. Solutions that did better identify *B* and *D* only did so when their effects were much larger than physically plausible, a priori values of joint friction. In simpler PID cases, estimates of damping tend to be far less reliable than, for instance, the proportional coefficients, so for these reasons together it may be inadvisable to rely on postural sway data and estimation approaches to specifically determine joint friction. Similar concerns may be directed toward the active feedback damping *D*, though the prior ISDDE literature does not assert as specific of a definition nor necessary theoretical boundaries.

Estimates of both process noise and measurement error were very close to their true values in every case, with only small upward bias proportional to the magnitude of the estimate for certain parameter sets (Figs 3 and 4). The standard deviation of measurement error that we chose to simulate was *σ* = .01 cm, twenty times the error of the force plate used by Santos et al. [20] to obtain the data. The success of estimation despite greatly exaggerated sensor noise demonstrates the reliability of Kalman filtering and adequate technical specification of the model, and relieves researchers from the need to choose a preliminary noise reduction step such as spectral filtering. Instead, using the raw data and including measurement error in the model avoids removing fine-grained details of the signal represented in the domain of high frequencies typically suppressed by low-pass filtering.

The feedback delay, *τ*, was unbiased in noiseless simulations, and consistently biased downward in noisy simulations. It is not clear what causes the bias, but it did not appear to consistently induce bias in other parameters that depended on the correct lag interval, such as the active proportional and derivative controllers.

The results of our simulation demonstrate that the proposed method of direct, statistical estimation by Kalman filter can recover the complete set of parameters for the model. Previous estimation methods only attempted to estimate five of the eight structural parameters [17, 18]. Among those attempted, the *D* parameter did not converge to its true value in simulation nor to a reliable, unimodal distribution in the empirical study. Despite this setback, no discussion was given of the role of empirical identification in determining *D* or other parameters, whereas we have demonstrated that the precision of estimates depends on their true values and interdependence. Additionally, the accuracy of their results rests on assumed values of *K*, *B*, and *r*. Due to the high degree of parameter dependence in univariate models such as this, error in one parameter is expected to propagate to other parameters in a compensatory manner. It is therefore preferable to jointly estimate all uncertain model parameters when possible.

The prior studies also did not account for measurement error. We determined that additional sources of sensor noise could be filtered simultaneously with estimation of the dynamic structure. If additive noise is Gaussian, then no preprocessing steps such as spectral filtering or downsampling should be needed and the risk of obscuring important, fine-grained topological features is greatly mitigated.

Computationally, the use of global optimization to maximize the likelihood function provided an efficient alternative to Bayesian MCMC methods as no data simulation procedures, prior distributions, or posterior sampling were required. An additional, unexplored benefit of maximum likelihood in this case is estimation of standard errors directly from the likelihood function. Because the model includes discrete thresholds, the likelihood function was stochastic and non-differentiable. This prevented the use of the Hessian matrix to calculate precision. However, future work may explore methods of smoothly approximating the marginal likelihood function, for instance by fitting splines to likelihood values retained from the optimization procedure.

### Experimental data

The results of analyzing the empirical COM data show that the nonlinear mechanisms of feedback activation led to significant improvement in model fit over the simpler SDDE and SDE (i.e., delayed and instantaneous PID) models. It cannot be determined from statistical model comparisons alone whether the results validate the model-generating theory of posture control. To that end, we must compare the parameter estimates to their theoretical priors.

Overall, the distributions of parameters showed a feasible correspondence to the domains expected given the theory. *K* was consistently close to the 91% relative resistance found by Loram and Lakie [22] for all of the models tested, here showing resistance to 92% of the total gravitational toppling torque on average. Conversely, in the ISDDE and SDDE, *B* most often converged to zero and was not likely to play a critical role in the model behavior. Perhaps coincidentally, the mean of *B* was near its proposed value of 4 Nms/rad. It is possible that statistical power at the individual level was insufficient to identify small effects due to *B*, and the expected value would be recovered if it were estimated across the total data set. Active feedback was generally weaker than hypothesized but still sufficient for stability. Estimates of *P* were closer to .1 than the proposed .25 [1], likely due in part to the greater resistance to toppling forces from values of *K* closer to the high end of their theoretical distribution. *D* played a large role in the dynamics of active control and resulted in non-negligible damping in many individuals. Values of *a* and *r* reflected greater control sensitivity than expected. *a* values around 75% to 80% assign a larger share of the phase space to active feedback, while smaller values of *r* indicate less tolerance to falling at the origin of sway. The mean estimate of *a* was higher than found by Tietäväinen et al. [18], which reported a control space closer to 64% in accordance with the analysis by Asai et al. [1]. We found a nearly identical distribution of the feedback delay, *τ*, to Tietäväinen et al. [18], ranging from 200 to 400 ms with a mean around 300 ms. Estimates of *σ*_*w*_ were an order of magnitude smaller than expected by Asai et al. [1], and about half of those found by Tietäväinen et al. [18].

**Fig 10:**
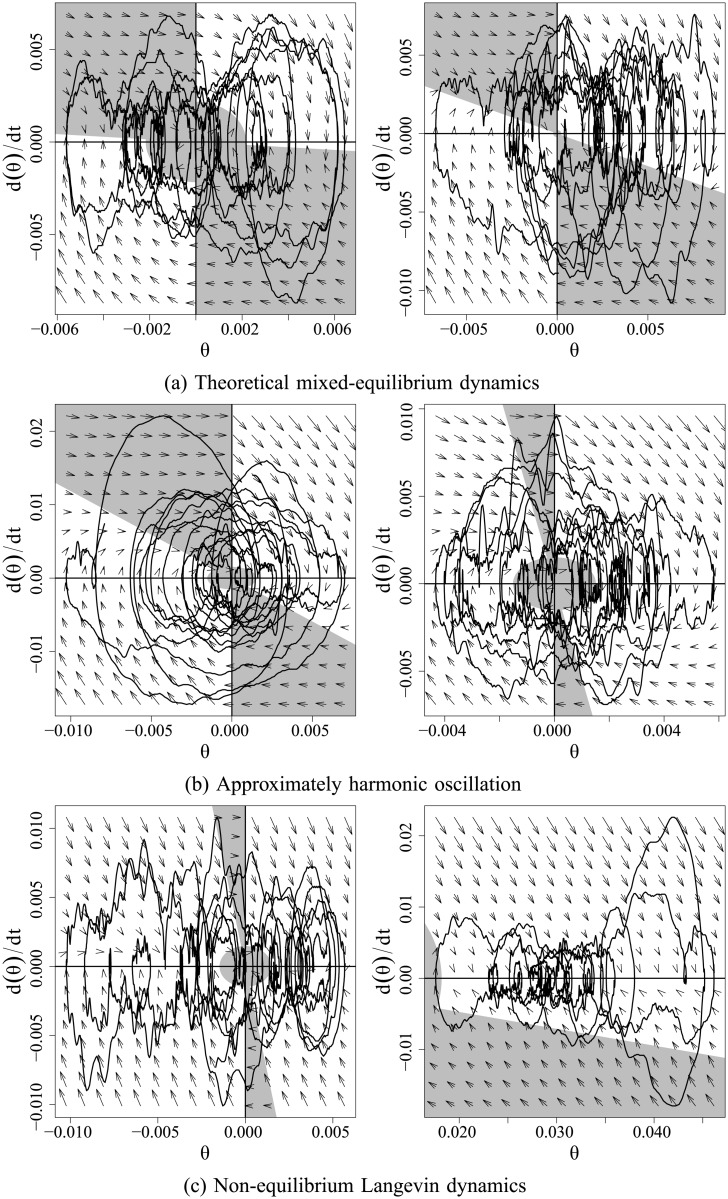
Phase portraits of observed body tilt angle series with estimated intermittent activation structures and vector fields. The horizontal axis is the COM or tilt angle position and the vertical axis is its velocity. The shaded region represents behavior where *P* and *D* are equal to zero. In (a), the estimated parameters match theoretical expectations, showing mixed equilibrium behaviors. In (b) and (c), the nonlinear mechanisms are fit in unique ways that deviate from their theoretically expected function.

A graphical vignette of these results is provided in Fig 10, which shows six raw data series with their respective intermittent activation conditions estimated by the model. The horizontal axis is the tilt angle and the vertical axis is the tilt angle’s velocity. The shaded region represents behavior where *P* and *D* are equal to zero. In the unshaded region, all parameters are active with their non-zero values. Fig 10a shows two cases that resemble the theorized structure with combinations of stable and unstable manifolds in nearly equal proportion. In Fig 10b, the values of *B* and *a* are sufficiently large to minimize the influence of the unstable manifold. The result is behavior that closely resembles harmonic oscillation around a single equilibrium. The opposite trend is shown in Fig 10c, where the unstable manifold is not influential, but a high ratio of the derivative coefficients to proportional coefficients results in continual suppression of velocity. This pattern results in wandering oscillations without clear equilibria.

**Fig 11:**
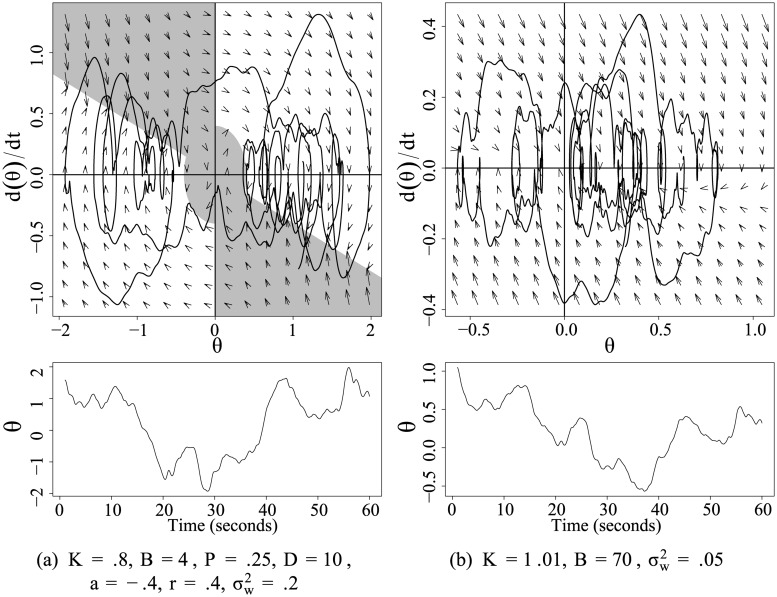
Phase portraits and time series from two models generated from the same vector of noise (scaled by *σ*_*w*_). In (a) data were simulated from the ISDDE model with the theoretical priors. In (b) data were simulated from a 3-parameter SDE. The large ratio of derivative (B) to proportional (K) force results in non-equilibrium Langevin dynamics that may exhibit similar features to the ISDDE for limited periods of time. Characteristic features distinguish (a) from (b), such as sharp changes in velocity localized to quadrants II and IV, slow change in velocity associated with quadrants I and III, and higher density at two spatial equilibria.

The optimal solution for the SDE model had a much larger, positive value for *B* than the other models while maintaining a theoretically plausible value of *K* less than 1. Furthermore, all values of *B* in the SDE were positive, as we might expect given that negative values would result in instability. When a linear system is strongly overdamped (in this case, a high value of *B* in the SDE, or either *B* or *D* in the SDDE) with relatively weak proportional feedback, as the SDE, then it exhibits non-equilibrium Langevin dynamics. These dynamics have conventionally described the random walk of a large molecule due to its collisions with a many smaller molecules in a solvent. The resulting trajectories can appear locally stationary by chance and exhibit short intervals of oscillation. Previous studies have modeled posture control in the context of Langevin dynamics [34–37]. Our simplest model of COM movement, the SDE, resembled a model of COP proposed by Bosek et al. [34] that describes trajectories as a second order SDE with no proportional feedback and a large derivative coefficient *B*. Fig 11 shows how the theoretical model and Langevin dynamics differ markedly in their mechanistic parameterization and observed phase portraits, yet they share many notable features. In both, high-frequency oscillations move gradually across the sample space in a “rambling” pattern. By chance, the Langevin equation in Fig 11b can result in concentrated oscillations around a few apparent equilibria, but no equilibrium mechanism is present in the model. The parsimony of generating these patterns with only three parameters poses a challenge to the specificity of evidence for the theoretical ISDDE. Visual inspection of the complete results showed that trials ranged between the two extremes of theoretical misspecification, from harmonic oscillation to Langevin dynamics. The expected topology involves a mix of features from both, sometimes showing adherence to the principles of feedback switching with occasional deviations into Langevin-type random walk.

Regardless of the true form of the underlying process, we might expect that if the parameters represent underlying physiological mechanisms, they should exhibit some degree of trait-like stability within-person. The intraclass correlations in Table 7 show that the nonlinear switching parameters were generally unreliable within-person. The correlations for the remaining parameters increase as the model is simplified to the SDDE and SDE. The higher consistency of the simpler models’ parameters does not necessarily imply that they are more “real” than those of the ISDDE. It is expected that reliance on fewer parameters to explain the variance of sway results in fewer competing configurations of those parameters. Any consistency of topological features within-person will be reflected in similarity of the model solutions. The lack of consistency in the more complex ISDDE is, however, a challenge to the trait-like stability and actuality of its parameters.

Though no specific connections between visual feedback and the theoretical mechanisms of control were hypothesized for the present study, we expected one or more parameters to be significantly influenced over trials in which eyes were closed in correspondence with previously observed effects on summary statistics. By modeling center of pressure variation with Langevin dynamics, Bosek et al. [34] found that the process noise distribution was influenced by visual feedback. The same finding was replicated with further connections to Parkinson’s disease [35]. Vieira et al. [38] found associations of visual feedback with stabilogram measures of sway. All three models models tested here had lower *p*-values for *σ*_*w*_ than for other parameters, suggesting that effects may be discernible given a larger sample or improvement in model specification.

Age has been previously associated with more general metrics of sway, such as path length [39], frequency band [38, 40] and mean velocity [40], though findings vary and few effects have been consistently reproduced in COP and COM data. Significant effects of age were observed in the present results, including ankle stiffness and active feedback force in the SDDE and process noise and ankle viscosity in the SDE. Interpretation of effects on the SDE is more difficult because the parameters of the SDE do not correspond to specific explanatory mechanisms in this case. The consistent positive associations of all models with noise magnitude *σ*_*w*_ with age may be linked to previously observed associations of stabilogram-based diffusion metrics with age [41]. The significant associations of ankle stiffness and active proportional feedback in the SDDE found here may reflect previously observed increases in stiffness and damping with age estimated from a simpler PID model [42].

Finally, the model concerns an abstract notion of body tilt angle, though there are many ways to represent this using the full kinematic data. For simplicity and consistency with past studies, we chose to represent tilt angle by the COM. Preliminary tests using alternative measures included COP and the average angle of both ankle joints. The results were found to differ markedly from both our current results and those previously obtained with the COM, but a complete comparison of alternative measures is too complex to discuss here. We leave detailed examination of this question with regard to the feasibility of this model to future study.

### Conclusions

We designed and implemented an Extended Kalman Filter-based estimation model of intermittent, delayed feedback control in postural sway and demonstrated that for a variety of stable configurations, parameters can be recovered accurately given adequate empirical identification. Application of the model to experimental data resulted in distributions of the parameters the correspond well to previous findings and suggest that physiologically informative and clinically useful attributes of human balance may be extracted directly from COM data. While the model replicates previous findings, the conjectured parameters of feedback activation were not reliable within-person or strongly associated with visual feedback and age. Further comparisons with alternative mechanistic theories and model parameterizations are warranted. Beyond postural control, the model stands as a framework for estimating parameters of stochastic delay differential equation models controlled by discrete activation thresholds.
